# Analysis of A Disintegrin and Metalloprotease 17 (ADAM17) Expression as a Prognostic Marker in Ovarian Cancer Patients Undergoing First-Line Treatment Plus Bevacizumab

**DOI:** 10.3390/diagnostics12092118

**Published:** 2022-08-31

**Authors:** Marina Fabbi, Delfina Costa, Daniela Russo, Laura Arenare, Gabriele Gaggero, Simona Signoriello, Giovanni Scambia, Carmela Pisano, Nicoletta Colombo, Nunzia Simona Losito, Gilberto Filaci, Anna Spina, Daniela Califano, Giosuè Scognamiglio, Angiolo Gadducci, Delia Mezzanzanica, Marina Bagnoli, Silvano Ferrini, Vincenzo Canzonieri, Paolo Chiodini, Francesco Perrone, Sandro Pignata

**Affiliations:** 1 UO Bioterapie, IRCCS Ospedale Policlinico San Martino, 16132 Genoa, Italy; 2 UO Oncologia Molecolare e Angiogenesi, IRCCS Ospedale Policlinico San Martino, 16132 Genoa, Italy; 3 Microenvironment Molecular Targets Unit, Istituto Nazionale Tumori IRCCS Fondazione G. Pascale, 80131 Naples, Italy; 4 Clinical Trials Unit, Istituto Nazionale Tumori IRCCS Fondazione G. Pascale, 80131 Naples, Italy; 5 UO Anatomia Patologica Ospedaliera, IRCCS Ospedale Policlinico San Martino, 16132 Genoa, Italy; 6 Department of Mental Health and Public Medicine, Section of Statistics, Università degli Studi della Campania Luigi Vanvitelli, 80131 Naples, Italy; 7 Department of Women and Child Health, Division of Gynecologic Oncology, Fondazione Policlinico Universitario A. Gemelli IRCCS, 00168 Rome, Italy; 8 Department of Life Science and Public Health, Catholic University of Sacred Heart, Largo Agostino Gemelli, 00168 Rome, Italy; 9 Urogynecological Medical Oncology, Istituto Nazionale Tumori IRCCS Fondazione G. Pascale, 80131 Naples, Italy; 10 European Institute of Oncology IRCCS, University of Milan-Bicocca, 20126 Milan, Italy; 11 Pathology Unit, Istituto Nazionale Tumori IRCCS Fondazione G. Pascale, 80131 Naples, Italy; 12 Department of Internal Medicine, University of Genoa, 16132 Genoa, Italy; 13 Department of Clinical and Experimental Medicine, Division of Gynecology and Obstetrics, University of Pisa, 56127 Pisa, Italy; 14 Molecular Therapies Unit, Department of Research, Fondazione IRCCS Istituto Nazionale dei Tumori, 20133 Milan, Italy; 15 Pathology Unit, Centro di Riferimento Oncologico di Aviano (CRO) IRCCS, 33081 Aviano, Italy; 16 Department of Medical, Surgical and Health Sciences, University of Trieste, 34127 Trieste, Italy

**Keywords:** ovarian cancer, ADAM17, immunohistochemistry, bevacizumab treatment, prognostic biomarker

## Abstract

To find prognostic factors for advanced ovarian cancer patients undergoing first-line therapy with carboplatin, paclitaxel and bevacizumab, we investigated the expression of a disintegrin and metalloprotease 17 (ADAM17) in cancer tissues. ADAM17 has been involved in ovarian cancer development, progression and cell resistance to cisplatin. Tissue microarrays from 309 ovarian cancer patients enrolled in the MITO16A/MANGO-OV2 clinical trial were analyzed by immunohistochemistry for ADAM17 protein expression. Intensity and extent of staining were combined into a semi-quantitative visual grading system (H score) which was related to clinicopathological characteristics of cases and the clinical outcome of patients by univariate and multivariate Cox regression models. ADAM17 immunostaining was detected in most samples, mainly localized in the tumor cells, with variable intensity across the cohort. Kaplan–Meier survival curves, generated according to the best cut-off value for the ADAM17 H score, showed that high ADAM17 expression was associated with worse prognosis for PFS and OS. However, after the application of a shrinkage procedure to adjust for overfitting hazard ratio estimates, the ADAM17 value as prognostic factor was lost. As subgroup analysis suggested that ADAM17 expression could be prognostically relevant in cases with no residual disease at baseline, further studies in this patient category may be worth planning.

## 1. Introduction

Ovarian cancer is the most lethal gynecological malignancy, mostly diagnosed at an advanced stage, when the standard treatment is a combination of surgery and platinum-based chemotherapy [1,2]. In the last decade, the therapeutic options in the first-line treatments of advanced ovarian cancer have been implemented by the combination of carboplatin/paclitaxel with the anti-vascular endothelial growth factor (VEGF) antibody bevacizumab [3,4,5,6], and with the recent addition of the poly ADP-ribose polymerase (PARP) inhibitor olaparib [7]. In all instances, with the exception of a BRCA mutation driving the use of PARP inhibitors [8,9,10], prognostic/predictive biomarkers for clinical use are still an unmet need. The MITO16A/MaNGO-OV2 (EudraCT number: 2012-003043-29, hereafter indicated as MITO16A) clinical trial was aimed to evaluate clinical and biological prognostic factors for advanced ovarian cancer patients receiving first-line treatment with carboplatin, paclitaxel and bevacizumab [11].

A disintegrin and metalloprotease 17 (ADAM17) is involved in cancer development and progression due to its ability to shed many substrates involved in inflammation, growth factor receptor activation, angiogenesis, inter-cellular adhesion, drug-resistance and immune regulation, reviewed in [12,13,14,15,16,17]. ADAM17 proteolytically cleaves and activates several EGFR ligands such as epiregulin, transforming growth factor alpha (TGF-alpha), amphiregulin (AREG) and heparin-binding EGF-like growth factor (HB-EGF) and may thus contribute to cancer progression through EGFR activation [12,14,18,19,20,21]. In particular, the ADAM17 substrates AREG and TGF-alpha were found in the ascites of ovarian cancer patients, suggesting high activity of ADAM17 in this tumor [22,23]. In addition, a monoclonal antibody blocking ADAM17 activity inhibited shedding of the ADAM17 substrates TNF, TNFR1-alpha, TGF-alpha, AREG, HB-EGF and IL-6Ralpha and reduced tumor growth, in an ovarian cancer xenograft model [24].

ADAM17 plays a role in the resistance of ovarian cancer cells to cisplatin. Indeed, treatment of ovarian cancer cells with cisplatin in vitro resulted in enhanced ADAM17 activity, shedding of AREG and triggering of EGFR signaling. Inhibition of ADAM17 with chemical inhibitors, anti-ADAM17 antibodies or gene silencing reduced AREG release and sensitized cancer cells to cisplatin-induced apoptosis [25].

ADAM17 activity also regulates tumor cell adhesion through the shedding of adhesion molecules, [13], including activated leukocyte cell adhesion molecule (ALCAM). Indeed, ALCAM is expressed at the cell surface of ovarian cancer cells, and its shedding by ADAM17 is involved in cell migration and invasion. Moreover, activation of ADAM17 by EGF increases ALCAM shedding by ovarian cancer cells and in vitro invasiveness. These effects are blocked by ADAM inhibitors or by ADAM17 gene silencing, further suggesting that ADAM17 may represent a therapeutic target in EGF-dependent tumors [26].

Finally, ADAM17 supports pathological neovascularization in different conditions, including tumors. In cancer, the pro-angiogenic role of ADAM17 is related to the shedding and activation of different pro-angiogenic factors such as HB-EGF [27], neuregulin-1 [28] or TGF-alpha [29] or, conversely, to the downregulation of the natural inhibitor of angiogenesis thrombospondin-1 (TSP1) [30]. Moreover, ADAM17 activity promotes the migration and sprouting of lymphatic endothelial cells through the activation of EGFR ligands [31]. Altogether these findings suggest that ADAM17 may support VEGF-alternative pathways for neo-angiogenesis in cancer.

In view of the possible role of ADAM17 in ovarian cancer, in cisplatin resistance and in the induction of angiogenesis pathways alternative to VEGF, in this study we investigated the ADAM17 protein expression by immunohistochemistry (IHC) in tissue microarrays (TMAs) from the cohort of ovarian cancer patients enrolled in the MITO16A/MANGO-OV2 clinical trial. The different expression levels of ADAM17, in terms of both staining intensity and percent of positive cells, were combined in a final score and associated to patient clinicopathological characteristics and clinical prognosis.

## 2. Materials and Methods

This study is part of the translational activities associated with the MITO16A/MaNGO-OV2 (EudraCT number: 2012-003043-29) clinical trial, coordinated by the Clinical Trials Unit at Istituto NazionaleTumori IRCCS “Fondazione G. Pascale” in Naples. MITO16A, a multicenter, phase IV, single-arm trial of bevacizumab in combination with carboplatin and paclitaxel was designed with the search for prognostic biomarkers as one of its primary endpoints [11]. Briefly, main eligibility criteria for patient enrollment were: FIGO stage IIIB–IV, previously untreated epithelial ovarian cancer, performance status 0–2 according to ECOG (Eastern Cooperative Oncology Group), no contraindications to bevacizumab administration, adequate organ function, no history of other tumors or mild to severe cardiovascular disease. Availability of tumor samples for molecular analyses was mandatory. The study was conducted in accordance with the ethical standards and according to the Declaration of Helsinki and national and international guidelines. All patients signed an informed consent form before entering the trial. The study was approved by ethical committees at each participating center.

### 2.1. Immunohistochemistry

FFPE block collection, pathological revision and processing for tissue microarrays (TMAs) building is thoroughly described in reference [32]. Briefly, in the MITO16A clinical trial, the FFPE blocks collection was prospective and not retrospective, ensuring material from 385 out of the 398 enrolled patients. The FFPE block from the primary tumor was the preferential site requested for translational analyses, but, when this was not available, a block from synchronous peritoneal secondary localization was used. Moreover, three cores from each patient were enclosed in the TMAs, thus addressing both the issue of tumor heterogeneity and the risk of sample loss. TMA sections, 5 µm thick, were cut and sent to the recipient lab, where samples were processed for ADAM17 staining within 2 weeks of reception. Immunohistochemistry (IHC) was carried out by an automated Bond-RX immunostainer (Leica, Wetzlar, Germany). The seven slides carrying the TMAs from the MITO16A cohort were all processed simultaneously. As anti-ADAM17 antibody, we chose HPA010738 antibody produced in rabbits (Sigma-Aldrich, Prestige Antibodies^®^ Powered by Atlas Antibodies), which is validated for use on paraffin-embedded sections by The Human Protein Atlas (https://www.proteinatlas.org; accessed on 2 September 2019). The antigen retrieval method was HIER pH6, according to manufacturer’s instructions. Anti-ADAM17 primary antibody was used at a 1:50 dilution and detected by the Bond Polymer Refine Detection kit (Leica Biosystems, Buccinasco, Italy). Slides were digitalized by an Aperio AT2 scanner (Digital Whole Slide Scanning, Leica Biosystems) at 20× and 40× magnification.

### 2.2. Evaluation of IHC Staining

Two observers (G.G., a pathologist, and M.F., experienced in IHC) evaluated the sections and scored each TMA core. Both were blinded to the clinicopathological parameters and clinical outcomes of the patients. Discrepant scores between the two observers, reported in less than 5% of cases, were discussed to achieve a consensus. ADAM17 expression was quantified by a semi-quantitative visual grading system, which combined the extent of staining (percent of immunoreactive tumor cells) and the staining intensity, visually scored and stratified as: 0 (negative), 1 (weak), 2 (moderate) and 3 (strong). A final score, defined as the H score, was assigned using the following formula: H = [1 × (% cells 1) + 2 × (% cells 2) + 3 × (% cells 3)]. Therefore, the weighted score generated for each core ranged from 0 (0% of tumor cells stained) to 300 (100% of tumor cells stained at intensity 3). To minimize the impact of tumor heterogeneity, each clinical case was represented by three cores in the TMAs, and thus we used the mean of the three samples as the final value of H score to be associated with patient characteristics and outcomes.

### 2.3. Statistical Analysis

Continuous variables were described with median values and interquartile range (IQR) or mean and standard deviation (sd), and qualitative variables were expressed in terms of absolute numbers and relative frequency.

Only patients with ADAM17 evaluation were analyzed and a histogram was used to describe the distribution of biomarkers.

Association between primary tumor and synchronous peritoneal secondary localization was evaluated by Pearson correlation coefficient with 95% confidence interval (CI) and by Bland–Altman plot in patients with both values.

The associations between ADAM17 and the clinical prognostic factors were investigated using the Wilcoxon rank sum test for dichotomous variables and the Kruskal–Wallis for categorical variables. The prognostic effect of ADAM17 was tested using progression-free survival (PFS) and overall survival (OS) as endpoints. PFS was defined as the time elapsing from the inclusion into the study to the first occurrence of either death for any cause or disease progression. OS was defined as the time elapsing from the inclusion into the study and death for any cause.

Kaplan–Meier curves were drawn for PFS and OS and compared with a two-sided log-rank test.

To test the prognostic role of ADAM17 on both PFS and OS, univariate and multivariate Cox proportional models were performed.

In the first univariate analysis, ADAM17 was tested as continuous variable after testing the linearity assumption using fractional polynomial. In a second univariate analysis, the biomarker was tested as a categorical variable searching for the best cut-off, selected by choosing the biomarker value that minimized the p-value of hazard ratio (HR) for the categorical variable defined by the cut-off value. The best cut-off search was calculated on PFS and then applied to the OS. To adjust for overfitting HR estimates of best cut-off categories, a shrinkage procedure with 95% bootstrap percentile method CI was performed [33].

A multivariable analysis was performed using as covariates: age (as category <65 vs. ≥65), ECOG performance status (PS) (0 vs. 1–2), residual disease (None; ≤1 cm; >1 cm; not operated), FIGO stage (III vs. IV), tumor histology (high-grade serous vs. other) and ADAM17 (considered as continuous variable and as categorical variable defined by best cut-off value).

Exploratory subgroup analyses for PFS and OS were performed and reported in a forest plot including p-values for the first-order interactions between ADAM17 and covariates, tested with the likelihood-ratio test of two nested models, with and without interaction.

Data were analyzed using R software version 3.6.0 (R Foundation for Statistical Computing, Vienna, Austria).

## 3. Results

### 3.1. Expression of ADAM17 Protein in Ovarian Cancer Tissue Microarrays (TMAs)

Expression of the ADAM17 protein was investigated by IHC in TMAs from the MITO16A clinical trial. Out of 398 patients enrolled in centers contributing to translational studies, 89 cases were excluded due to inadequate, exhausted or not available tumor samples or to sample collection during interval debulking surgery after neo-adjuvant chemotherapy (Supplementary Figure S1). A total of 309 cases were thus available for analysis of ADAM17 expression. Among these, in 234 cases the cancer tissue in TMAs was obtained from the primary localization in the ovary, whereas in 75 cases only samples from synchronous peritoneal secondary localization could be used. In 45 of the 234 cases with tissue from primary localization, we could also include in TMAs tissue from synchronous secondary localization, which allowed us to compare paired “primary” versus “metastasis” samples. Table 1 shows the baseline characteristics of patients and disease of the 309 cases available for this study as compared to the total MITO16A population. As shown, the subset of patients in TMAs is largely composed of high-grade serous carcinomas (85.8%) and is representative of the MITO16A population with a slight increase in patients with better prognosis related to residual disease.

**Figure 1:**
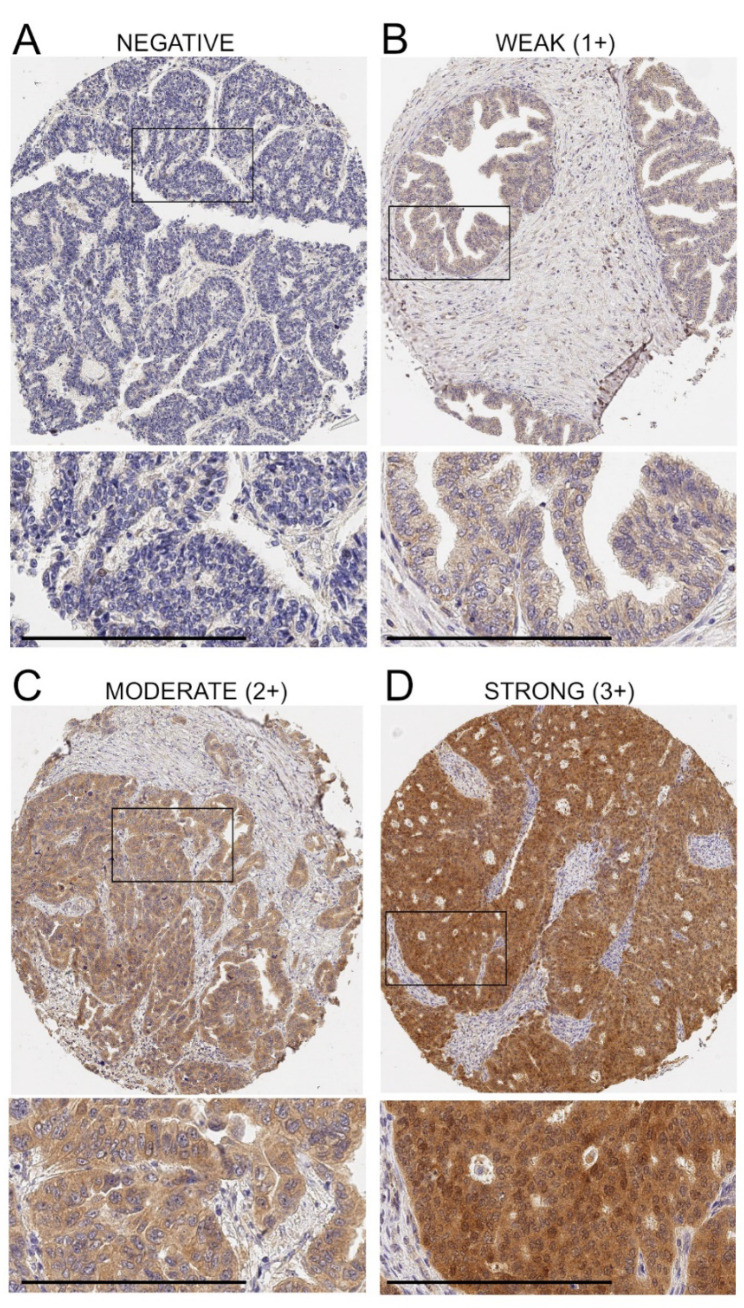
Variation of ADAM17 protein expression in ovarian cancer tissue. Representative examples of ADAM17-negative tissue (**A**) and weak (**B**), intermediate (**C**) and strong (**D**) immunostaining are shown. Bar = 200 μm. Enlarged areas are marked by insets. Once the staining intensity was integrated with the percent of positive cells, the final H score values generated for these cores were equal to 0 (**A**), 100 (**B**), 200 (**C**) and 240 (**D**).

The staining intensity that we observed for anti-ADAM17 antibody was variable across the cohort and the staining pattern was cytoplasmic and mainly localized in the tumor cells, whereas the stroma was negative (all the staining is provided in Supplementary Figure S2). Cores from normal fallopian tubes, seeded in our TMAs as controls, showed a weak staining for ADAM17 (Supplementary Figure S3), consistent with the low expression reported for epithelial tube tissue in the Human Protein Atlas (https://www.proteinatlas.org; accessed on 10 August 2022). To classify samples, we opted for a semi-quantitative visual grading system (H score), which combined the staining intensity, stratified as shown in Figure 1 by four representative cores, with the percent of immunoreactive tumor cells, as detailed in the methods section. An H score was thus obtained for each core. Additional examples of staining with the resulting H score values are shown in Supplementary Figure S4. As each clinical case was represented by three cores in the TMAs, to take into consideration the intra-tumor heterogeneity, we used the mean H score value of the triplicates in the subsequent analyses.

The distribution of ADAM17 immunostaining expressed as an H score in the MITO16A cohort is charted in Supplementary Figure S5. Out of 309 cases, only 37 (12%) were completely negative (0% of tumor cells stained). The median value for H score was 80.0 (IQR 33.3–126.7) and the mean (±sd) value was 86.3 (±66.8).

When we compared the ADAM17 immunostaining in the 45 paired primary and metastasis samples, we observed that the mean (±sd) value of H score was 73.8 (±57.9) in primaries versus 91.6 (±69.2) in metastases, with a P value = 0.0242 by paired Student’s *t* test. This observation suggests that ADAM17 expression may be higher in secondary peritoneal localization. However, this is not true for all the paired samples, as in several cases the metastasis H score was lower than that of the primary tumor. When we analyzed the relationship between H score in primaries and metastases we found a significant Pearson’s correlation (r = 0.689 95%CI: 0.495 to 0.817, *p* < 0.0001). However, when a Bland–Altman plot was applied, a low agreement was found (mean differences −18.1 95%CI (−118.3; 82.1)) (Supplementary Figure S6). Nevertheless, to not further weaken the power of the study, which had a pre-specified sample size of 400 patients, we decided to analyze the 234 primaries and 75 metastases as a global cohort for the evaluation of the performance of ADAM17 protein expression as prognostic marker in the MITO16A study.

### 3.2. Evaluation of ADAM17 Expression as Prognostic Marker

We first verified whether the level of expression of the ADAM17 protein, summarized in the H score, was related to any clinicopathological characteristics of the analyzed case material. No significant associations were found, although high grade tumors appeared to express higher levels of ADAM17 (Table 2).

In order to evaluate the prognostic effect of ADAM17 expression, we first analyzed its expression as continuous variable in univariable and multivariable Cox models for PFS (222 events) and OS (102 events). For both endpoints, no prognostic effect was found for ADAM17 expression (Supplementary Table S1); using fractional polynomial the final model for the biomarker was found to have only linear effects.

We then searched for the biomarker’s best cut-off value that minimizes the p-value of the HR (see methods for details). The best cut-off found was equal to ADAM17 H score at 50 (Supplementary Figure S7) and it was used to test the ability of the biomarker to predict patient prognosis for both PFS and OS.

**Figure 2:**
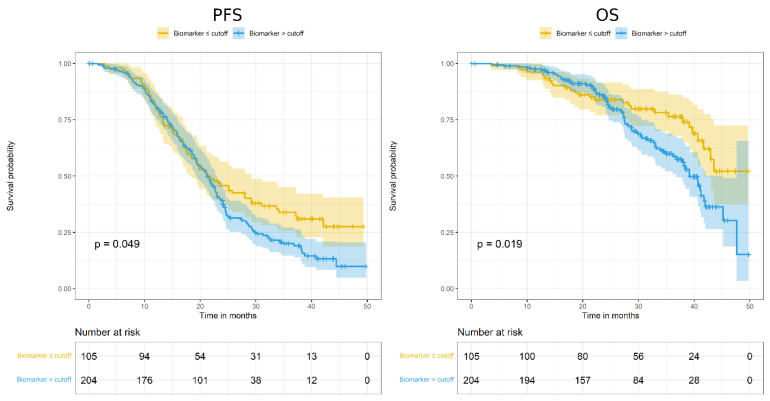
Kaplan–Meier curves assessing progression-free survival (PFS; **left**) and overall survival (OS; **right**) according to ADAM17 expression, as dichotomized around the cut-off value of H score = 50.

Analysis of ADAM17 H score as prognostic factor, as dichotomized according to best cut-off value, showed a prognostic effect on PFS and OS (Figure 2; Table 3).

In particular, for both endpoints, patients with lower ADAM17 H score showed better prognosis. However, these significant associations disappeared after HR adjustment for overfitting (Table 3).

We next tested the prognostic value of ADAM17 in a multivariable analysis adjusted for patient clinical characteristics. For both PFS and OS, no prognostic effect was found (Table 3).

**Figure 3:**
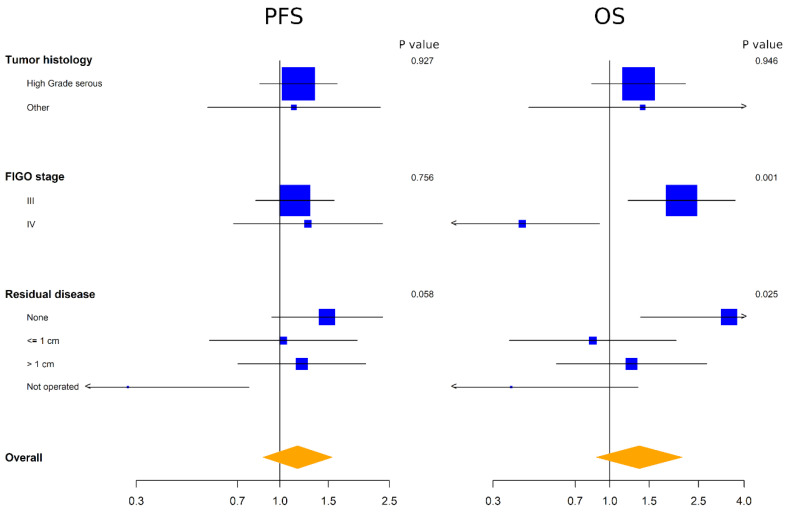
Forest plot representing exploratory subgroup analyses of the effect of ADAM17 expression for PFS (**left**) and OS (**right**). Side numbers are p values and bottom numbers are HR.

The heterogeneity of the prognostic effect of ADAM17 expression was also studied in a subgroup analysis of histology, FIGO stage and residual disease with the aim to generate hypotheses. No significant interactions were found on PFS. However, a trend was observed between ADAM17 expression and residual disease on PFS, where high ADAM17 levels were predictive of worse prognosis among patients without residual disease compared to patients with residual disease at baseline. This interaction was significant for OS, suggesting that high ADAM17 expression should be further explored as a negative prognostic factor in patients with no residual disease. At variance with results on PFS, a significant interaction was found on OS between ADAM17 and FIGO stage, where the high expression of ADAM17 was predictive of worse prognosis among patients with FIGO stage III compared to patients with FIGO stage IV at baseline (Figure 3).

## 4. Discussion

In this study we report the detection by IHC of ADAM17 protein expression in EOC tissue from a cohort of patients undergoing first-line therapy plus bevacizumab (MITO16A study) and its association with clinical outcomes. ADAM17 immunostaining was mainly localized in the tumor cell cytoplasm and was detected in most samples, with variable intensity across the cohort. ADAM17 levels of expression were not associated with clinical–pathologic characteristics of cases, and when related to PFS and OS, the association found between high ADAM17 expression and worse prognosis lost significance after HR adjustment for over-fitting.

Our finding that ADAM17 is widely expressed in ovarian cancer cells is in line with previous studies, which report that positive IHC staining of ADAM17 is observed in the cytoplasm of tumor cells with no staining in normal ovarian epithelium [34,35]. However, in the literature, the relationship between expression of ADAM17 and patient characteristics and outcomes has been addressed mainly by gene expression analysis [34,36,37,38]. These studies report that ADAM17 gene expression is significantly enhanced in both early and advanced ovarian cancer compared with that in normal ovaries [34] and that higher ADAM17 expression is associated with significantly decreased PFS of grade 1 and 2 serous ovarian cancer patients [36]. Systematic analysis of large data portals identifies the ADAM17 gene as one of the NOTCH-associated genes overexpressed in ovarian cancer tissues compared with normal ovaries, generally associated with advanced cancer stages and with poor PFS and OS times [38]. Moreover, long non-coding RNA CCAT1, that is upregulated and associated with poor prognosis in EOC, downregulates miR-152, thus triggering upregulation of ADAM17 and WNT1, with subsequent promotion of EMT [37]. Altogether these previous data suggested that increased ADAM17 mRNA expression associates with EOC progression. In addition, a recent study investigated ADAM17 protein in serum as a diagnostic biomarker in ovarian cancer. Interestingly, high ADAM17 concentrations in serum at primary diagnosis were associated with early FIGO I/II stages and with no residual disease, suggesting that ADAM17 can be a promising screening marker for early-stage high-grade serous EOC [39].

The expression of ADAM17 protein is here analyzed for the first time in an EOC population treated in first line with bevacizumab associated with carboplatin/paclitaxel. These IHC data, which record ADAM17 protein levels that can be differently regulated, cannot be directly compared with mRNA expression data but only to other studies using a similar approach. More IHC data are available from studies of ADAM17 expression in other cancer types. Strong expression of ADAM17 was found associated to poor prognosis or aggressive disease progression in gastric cancer [40,41,42,43], hilar cholangiocarcinoma [44], cervical [45], esophageal [46], non-small-cell lung cancers [47] and in high-grade breast tumors [48]. In colon cancer, ADAM17 protein is overexpressed in primary and metastatic tumors compared with normal colonic mucosa and the intensity of its immunoreactivity is inversely correlated with that of TGF-alpha and amphiregulin [49], with ADAM17 gene expression significantly higher in liver metastases than in primary tumors [50]. High ADAM17 expression was related to tumor invasiveness or adverse prognosis also in hepatocellular carcinoma [51], breast cancer [52], clear-cell renal carcinoma [53] and glioblastoma [54]. Although consistently indicating that ADAM17 plays a relevant role in cancer progression and outcome, these reports did not yield a consensus value that supported the use of ADAM17 as a biomarker. Indeed, most of the papers reporting IHC analyses of ADAM17 expression in tumor tissues employed an evaluation method based on a semiquantitative weighted histoscore, which combines staining intensity and percent of positive cells. However, these parameters were variously grouped and either summed [55] or multiplied [40,41,42,43,44,47,51,54], and a given score value was then chosen to split negative or low- from high-expression cases. In the present study, we adopted the evaluation method described by Gao et al. [56] and applied to ADAM17 immunostaining by Jiao et al. [44], which produces a weighted score for each case, ranging from 0 (0% of cells staining) to 300 (100% of 3+ strongly stained cells). Our reasoning was that a wide distribution of values could allow a better stratification of cases. Indeed, the calculation of the best cut-off value related to PFS found the score value of 50 as the one statistically significant. This value is close to the score <75 used to define ADAM17 negative/low expression by Jiao et al. [44], who reported that ADAM17 is overexpressed and is a poor prognostic indicator in hilar cholangiocarcinoma.

When we applied the H score cut-off value of 50 for ADAM17 to generate Kaplan–Meier survival curves in our cohort of patients, we found negative prognostic significance for high ADAM17 expression. This observation suggests that association of ADAM17 inhibition to this therapeutic schedule might improve efficacy. Indeed, a recent study reported that the combined in vitro treatment with an ADAM17 inhibitor and cisplatin showed enhanced cytotoxicity in ovarian cancer spheroids in comparison with cisplatin monotherapy, thus proposing ADAM17 as an interesting target for combinatorial treatments [57]. Moreover, a previous study in breast cancer model reported that ADAM17 was upregulated by severe hypoxia and xenografts that had been treated with bevacizumab showed significantly higher ADAM17 mRNA levels and enzyme activity compared with control tumors [58]. Therefore, if upregulation of ADAM17 has a role in supporting resistance to bevacizumab, the inhibition of its activity could be an additional target of combination therapies.

On the other hand, to identify strong prognostic biomarkers of survival, the MITO16A study was specifically designed with the application of a shrinkage procedure, aimed to adjust for over-fitting HRs estimates particularly when the best cut-off was used for biomarker dichotomization. With this statistical correction, the prognostic value of ADAM17 that we found in this case material was lost. As discussed in Califano et al. [59], where the same cohort was analyzed for the expression of angiogenesis-related genes as prognostic biomarkers, the application of a shrinkage procedure should be included in the design of more stringent statistical plans needed to define new biomarkers in oncology. However, it cannot be excluded that an increased sample size or an independent case material might provide a validation of our study.

Finally, with the aim of generating new working hypothesis, we performed a subgroup analysis of the effect of ADAM17 expression for PFS and OS. The finding that high ADAM17 expression effectively discriminated cases with higher hazard ratio in the subgroup with no residual disease at baseline, suggests that the role of ADAM17 in the course of carboplatin/paclitaxel plus bevacizumab therapy could be prognostically relevant only in this patient category. A significant prognostic effect of ADAM17 in FIGO stage III patients was found on OS but not in PFS, requiring new validation studies. Further investigations specifically designed to address these points are thus worth planning.

Among the limitations of our study, first we should mention the lack of information on the enzymatic activity of ADAM17 in the samples. Indeed, detection of ADAM17 by IHC does not discriminate between the inactive or enzymatically active state of the molecule. This aspect is relevant for the biological function of ADAM17 and could stratify cases in a different way. Secondly, the low prevalence in our cohort of histological sub-types other than high-grade serous carcinomas limits the generalization of the findings. Moreover, among the inclusion criteria of the MITO16A clinical trial was FIGO stage IIIB-IV. Therefore, early-stage cancers are totally absent in this study and we could have missed the population where ADAM17 could be a strong biomarker. Indeed, ADAM17 protein in serum has been reported as a potential blood-based biomarker for detection of early-stage ovarian cancer [39]. Last but not least, calculation of the H score and best cut-off value to dichotomize cases is not the only way to categorize ADAM17 expression, and different scoring methods could yield different results. Other scoring methods were described in the literature, but no consensus has been reached on a protocol and a value that supported the clinical use of ADAM17 as a biomarker, so far.

## 5. Conclusions

Analysis by Kaplan–Meier survival curves of ADAM17 immunostaining at first showed that low ADAM17 H score associated with better survival in ovarian cancer patients undergoing first-line treatment plus bevacizumab, suggesting that association of ADAM17 inhibition with this therapeutic schedule might improve efficacy. However, with the application of a shrinkage procedure to adjust for overfitting HR estimates as statistical correction, the prognostic value of ADAM17 lost significance. On the other hand, subgroup analysis indicated that ADAM17 expression could prognostically be relevant in cases with no residual disease at baseline, thus suggesting that further studies in this patient category should be planned.

## Figures and Tables

**Table 1:** Baseline characteristics of patients and disease of the cases analyzed as compared to the whole MITO16A cohort.

	Patients in Analysis	MITO16A Population
	(N = 309)	(N = 398)
**Median age (IQR)**	58.9 (49.8;66.2)	59.2 (49.8;66.5)
**Age category**		
<65	220 (71.2%)	278 (69.8%)
≥65	89 (28.8%)	120 (30.2%)
**ECOG performance status**		
0	248 (80.3%)	315 (79.1%)
1	53 (17.2%)	69 (17.3%)
2	8 (2.6%)	14 (3.5%)
**Residual disease**		
None	130 (42.1%)	153 (38.4%)
≤1 cm	63 (20.4%)	72 (18.1%)
>1 cm	96 (31.1%)	120 (30.2%)
not operated	20 (6.5%)	53 (13.3%)
**FIGO stage**		
IIIb	29 (9.4%)	36 (9.0%)
IIIc	220 (71.2%)	275 (69.1%)
IV	60 (19.4%)	87 (21.9%)
**Tumor histology**		
High-grade serous	265 (85.8%)	333 (83.7%)
Low-grade serous	11 (3.6%)	13 (3.3%)
Endometrioid	9 (2.9%)	9 (2.3%)
Clear cell	8 (2.6%)	11 (2.8%)
Mucinous	2 (0.6%)	3 (0.8%)
Mixed	2 (0.6%)	4 (1.0%)
Other	12 (3.9%)	25 (6.3%)

**Table 2:** Association between ADAM17 expression and baseline characteristics of patients and disease.

	Median (IQR)	P
**Age category**		0.3417
<65	75.8 (31.7–123.3)	
≥65	83.3 (50.0–140.0)	
**ECOG performance status**		0.3169
0	78.3 (30.0–123.3)	
1–2	88.3 (50.0–133.3)	
**Residual disease**		0.2962
None	66.7 (26.7–123.3)	
≤1 cm	86.7 (45.0–125.0)	
>1 cm	93.3 (42.5–133.3)	
not operated	81.6 (35.0–131.7)	
**FIGO stage**		0.4144
IIIb	83.3 (33.3–125)	
IV	66.7 (28.3–131.7)	
**Tumor histology**		0.0884
High-grade serous	83.3 (33.3–133.3)	
Other	65.0 (13.3–103.3)	

**Table 3:** Univariate and multivariate analysis for ADAM17 (categorized on best cut-off value) for PFS and OS. Original and shrunken coefficients are reported.

	**Progression-Free Survival**							
	**Univariate Analysis**				**Multivariate Analysis**			
	**Original Coefficients**		**Shrunken Coefficients**		**Original Coefficients**		**Shrunken Coefficients**	
ADAM17 >50 vs. ≤50	HR (95%CI)	*p*	HR (95%CI)	*p*	HR (95%CI)	*p*	HR (95%CI)	*p*
	1.33 (1.00–1.77)	0.050	1.24 (0.67–2.29)	0.501	1.16 (0.86–1.56)	0.329	0.99 (0.42–2.35)	0.986
	**Overall Survival**							
	**Univariate Analysis**				**Multivariate Analysis**			
	**Original Coefficients**		**Shrunken Coefficients**		**Original Coefficients**		**Shrunken Coefficients**	
ADAM17 >50 vs. ≤50	HR (95%CI)	*p*	HR (95%CI)	*p*	HR (95%CI)	*p*	HR (95%CI)	*p*
	1.68 (1.08–2.60)	0.020	1.52 (0.47–4.94)	0.483	1.36 (0.86–2.13)	0.186	1.14 (0.25–5.15)	0.868

## Data Availability

No new data were created or analyzed in this study. Data sharing is not applicable to this article.

## Supplementary Materials

The following supporting information can be downloaded at: https://www.mdpi.com/article/10.3390/diagnostics12092118/s1, Figure S1: schematic representation of the pathway leading to the selection of the 309 cases used for analysis of ADAM17 protein expression; Figure S2: Overview of the seven TMA slides immunostained for ADAM17 expression; Figure S3: Evaluation of ADAM17 protein expression in three representative cores of normal Fallopian tube samples enclosed in the MITO16A TMAs; Figure S4: Representative examples of ADAM17 immunostaining expressed as H score values; Figure S5: Distribution of ADAM17 immunostaining in the MITO16A cohort; Figure S6: Bland–Altman plot of ADAM17 H score values in paired samples from primary tumor and synchronous peritoneal secondary localization; Figure S7: Best cut-off value for ADAM17 H score; Table S1: Univariate and multivariate analysis for ADAM17 (continuous variable) for PFS and OS.
