# Supplementary material for: Analysis of A Disintegrin and Metalloprotease 17 (ADAM17) Expression as a Prognostic Marker in Ovarian Cancer Patients Undergoing First-Line Treatment Plus Bevacizumab
Source: Diagnostics (Basel). 2022 Aug 31;12(9):2118. doi: 10.3390/diagnostics12092118 (PMC9498026; doi:10.3390/diagnostics12092118)
Supplement: Supplementary file 1 [file diagnostics-12-02118-s001.zip › diagnostics-1846459-supplementary.pdf]

SUPPLEMENTARY MATERIALS: Analysis of A Disintegrin And Metalloprotease 17 (ADAM17) expression as a prognostic marker in ovarian cancer patients undergoing first-line treatment plus bevacizumab

Marina Fabbi, Delfina Costa, Daniela Russo, Laura Arenare, Gabriele Gaggero, Simona Signoriello, Giovanni Scambia, Carmen Pisano, Nicoletta Colombo, Nunzia Simona Losito, Gilberto Filaci, Anna Spina, Daniela Califano, Giosuè Scognamiglio, Angiolo Gadducci, Delia Mezzanzanica, Marina Bagnoli, Silvano Ferrini, Vincenzo Canzonieri, Paolo Chiodini, Francesco Perrone and Sandro Pignata

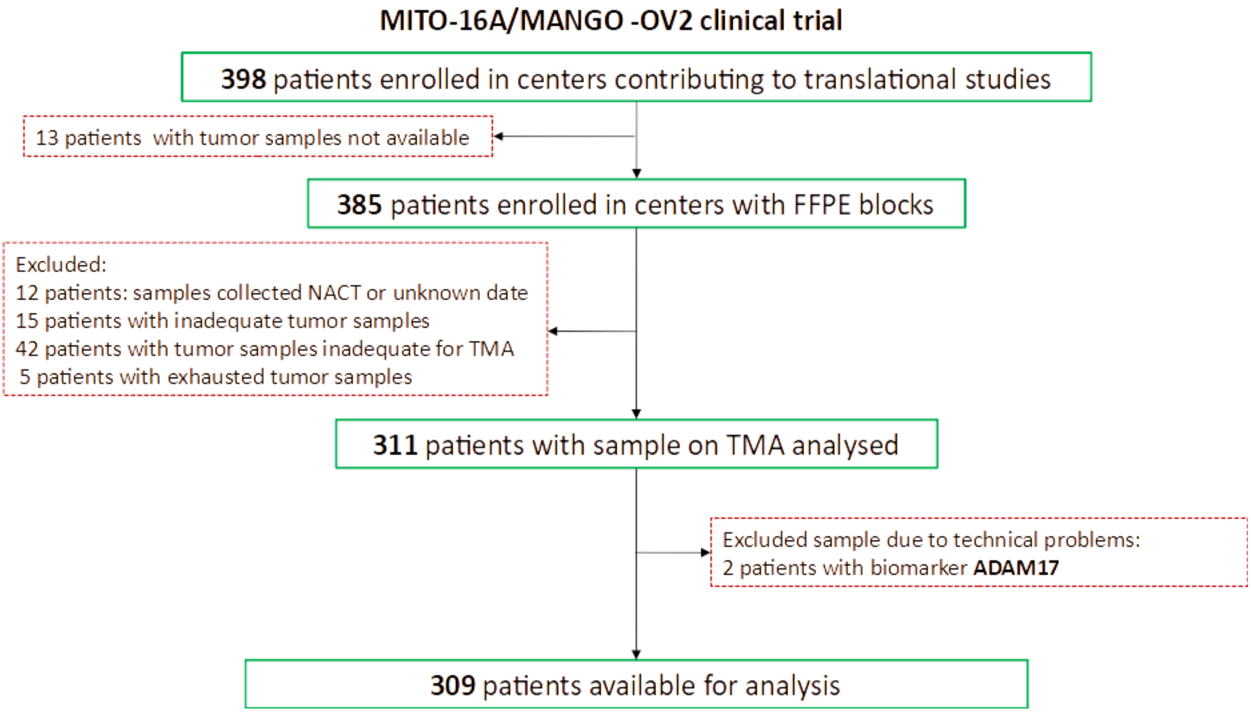

Figure S1. Schematic representation of the pathway leading to the selection of the 309 cases used for analysis of ADAM17 protein expression.

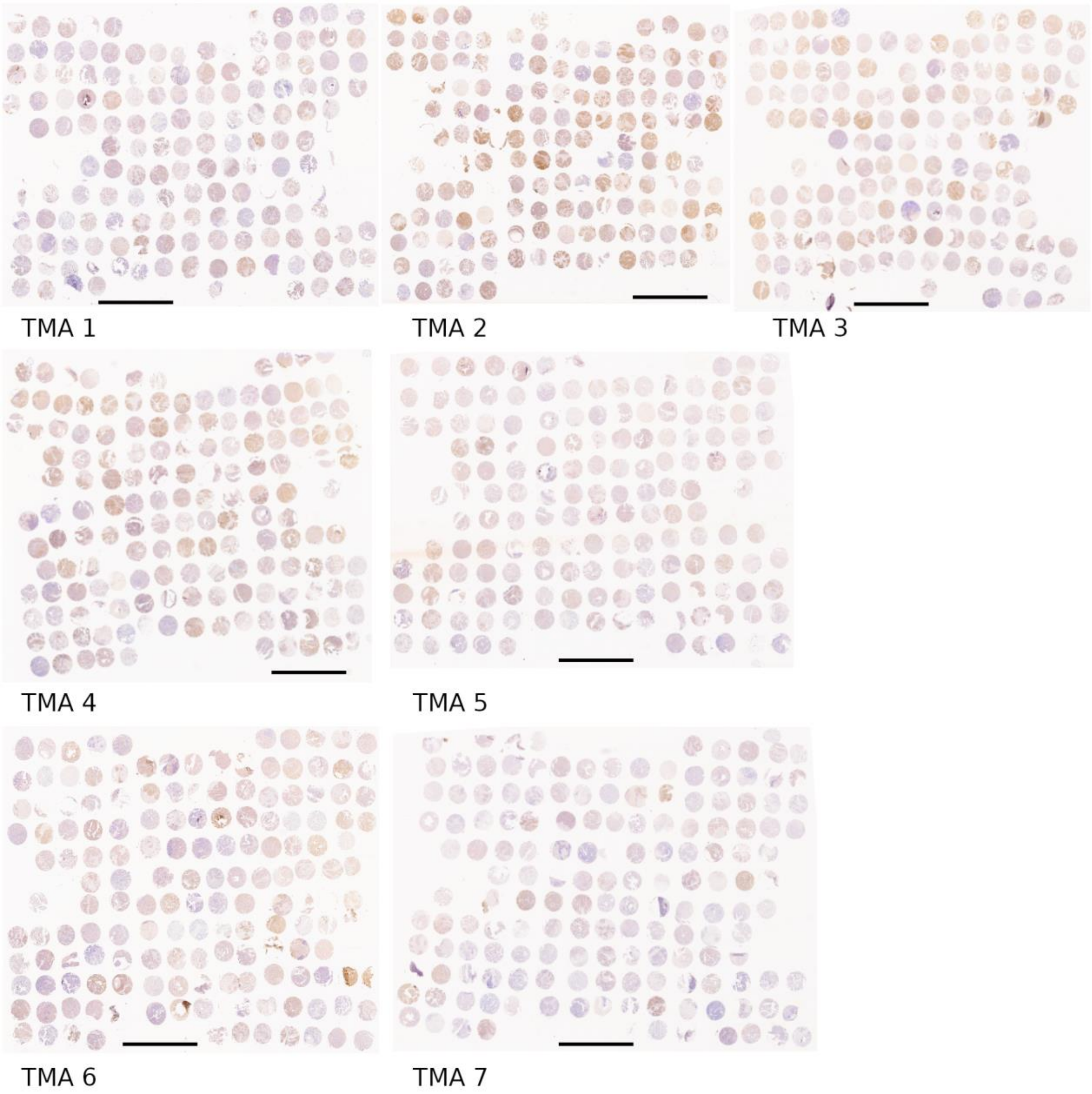

Figure S2. Overview of the seven TMA slides immunostained for ADAM17 expression. The staining intensity for anti-ADAM17 antibody is variable across the MITO16A cohort. Three cores from each MITO16A patient were enclosed in the TMAs, to overcome both tumor heterogeneity and the risk of sample loss. Bar 4 mm.

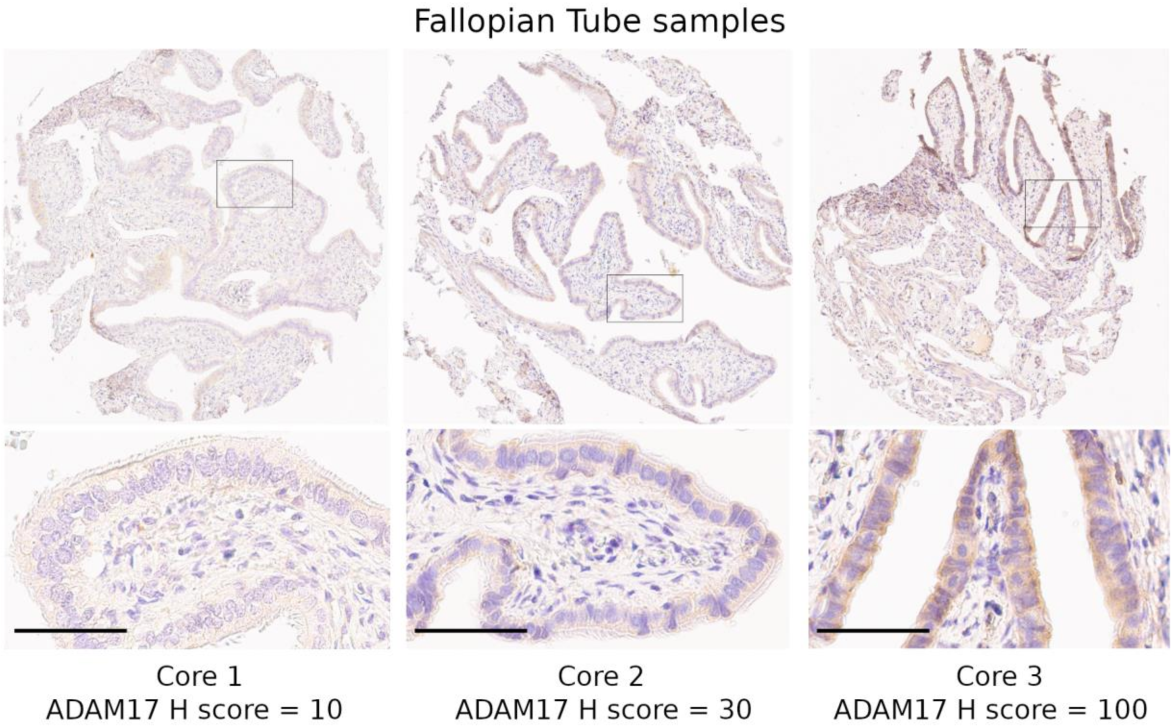

Figure S3. Evaluation of ADAM17 protein expression in three representative cores of normal Fallopian tube samples enclosed in the MITO16A TMAs. The corresponding H score values for ADAM17 expression are indicated. The highest value observed for ADAM17 in normal Fallopian tube samples was H score = 100. Bar 60 micrometers.

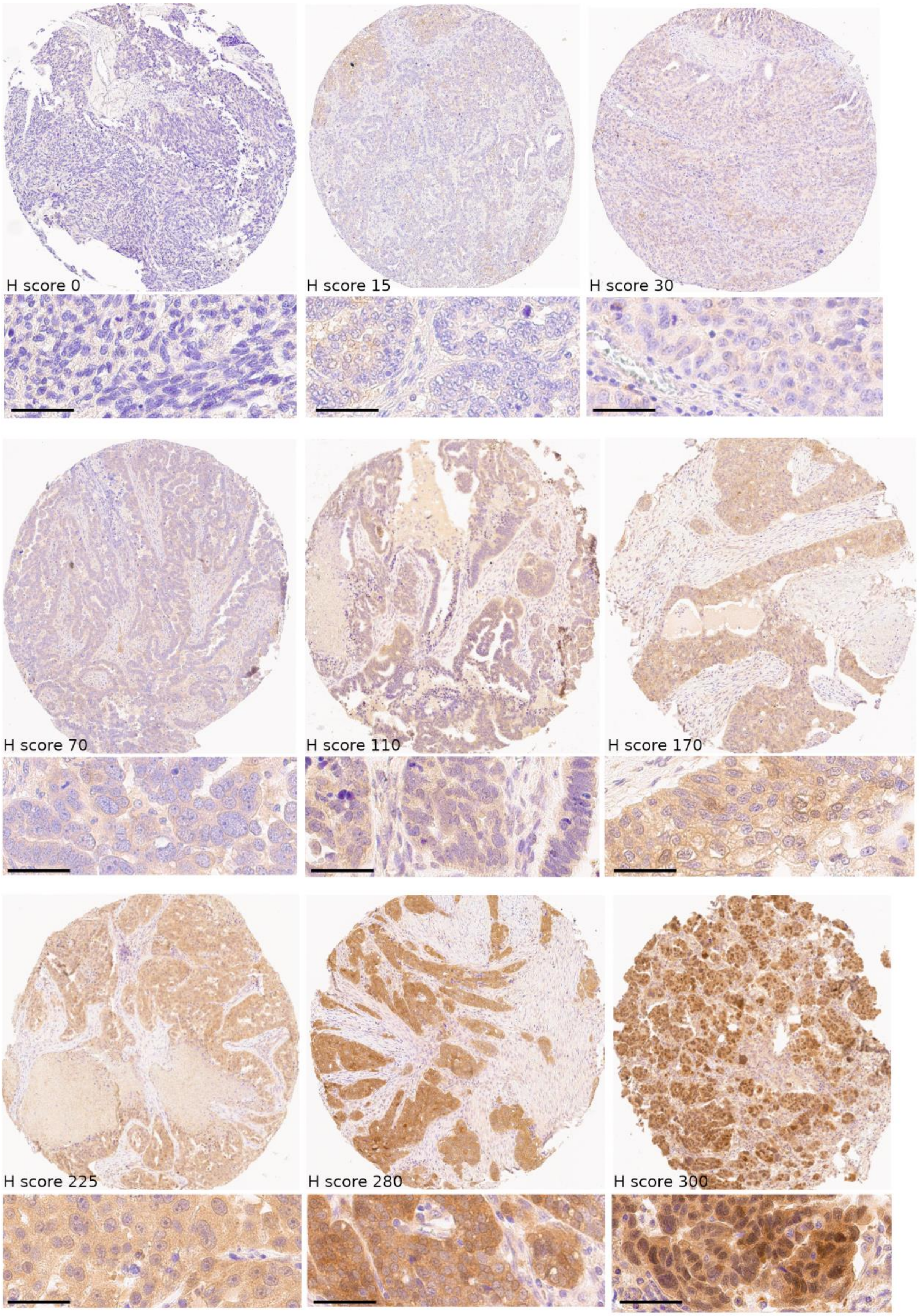

Figure S4. Representative examples of ADAM17 immunostaining expressed as H score values, which combine staining intensity and percent of positive tumor cells. Bar 60 micrometers.

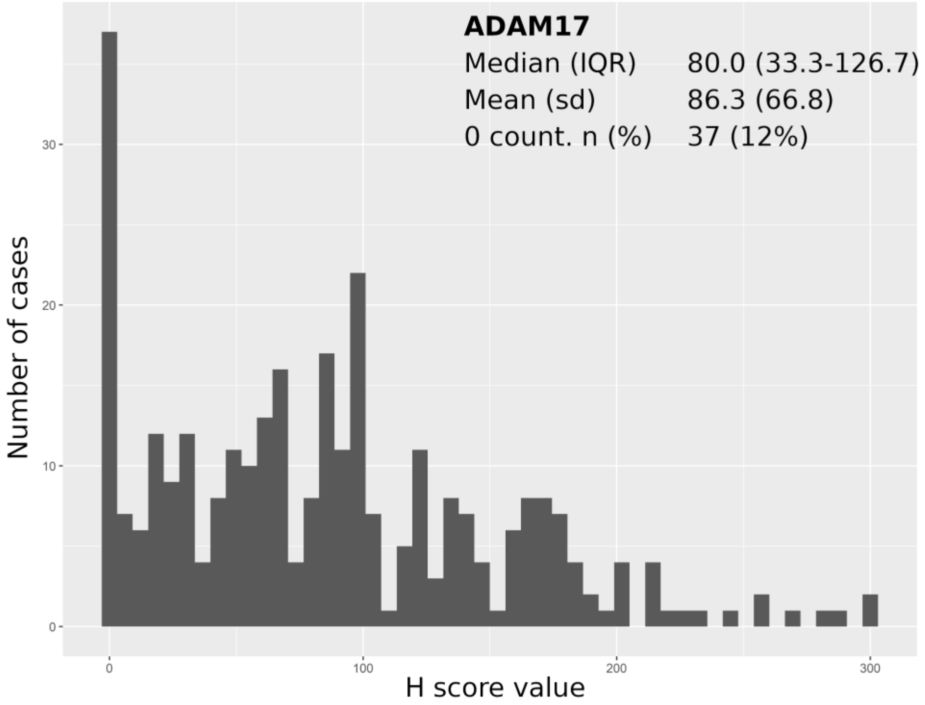

Figure S5. Distribution of ADAM17 immunostaining in the MITO16A cohort (n. 309). X axis: H score value; Y axis: number of cases.

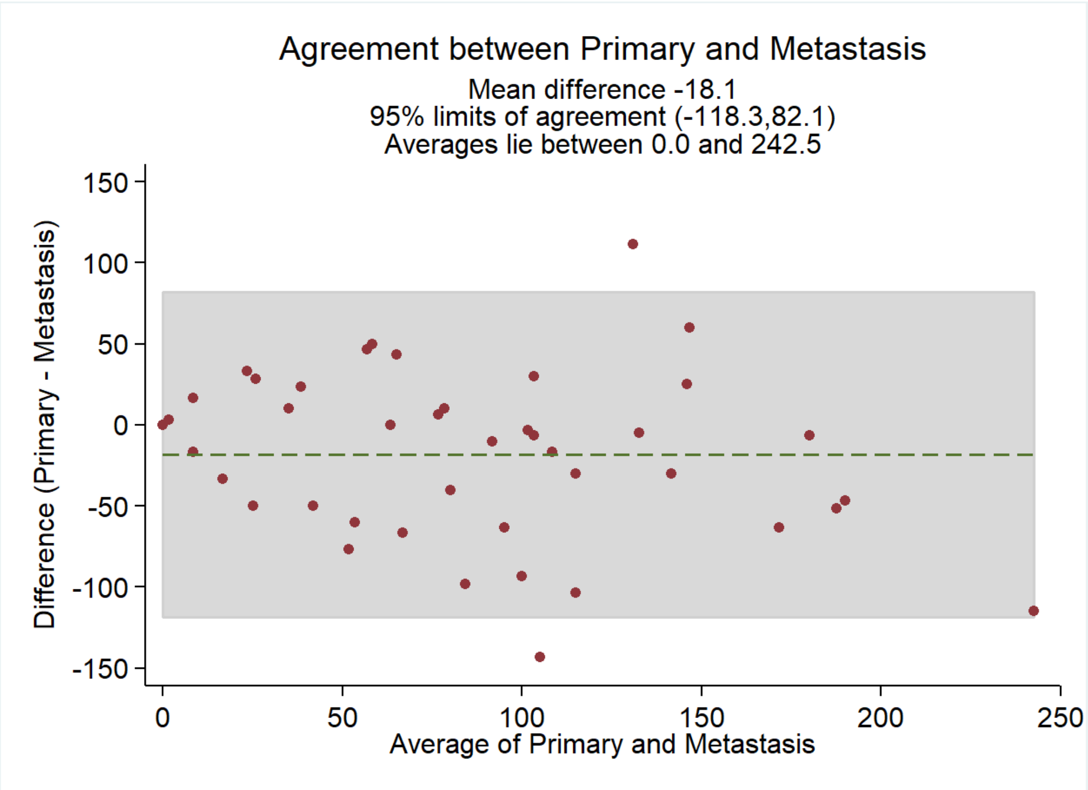

Figure S6. Bland and Altman plot of ADAM17 H score values in paired samples from primary tumor and synchronous peritoneal secondary localization. Number of pairs = 45.

Table S1. Univariate and multivariate analysis for ADAM17 (continuous variable) for PFS and OS

	**Progression Free Survival**				**Overall Survival**			
	Univariate analysis		Multivariate analysis		Univariate analysis		Multivariate analysis	
	HR(95%CI)	P	HR(95%CI)	P	HR(95%CI)	P	HR(95%CI)	P
**ADAM17** increment of 10	1.01(0.99-1.03)	0.337	1.01(0.99-1.03)	0.522	1.01(0.98-1.04)	0.362	1.01(0.98-1.04)	0.708

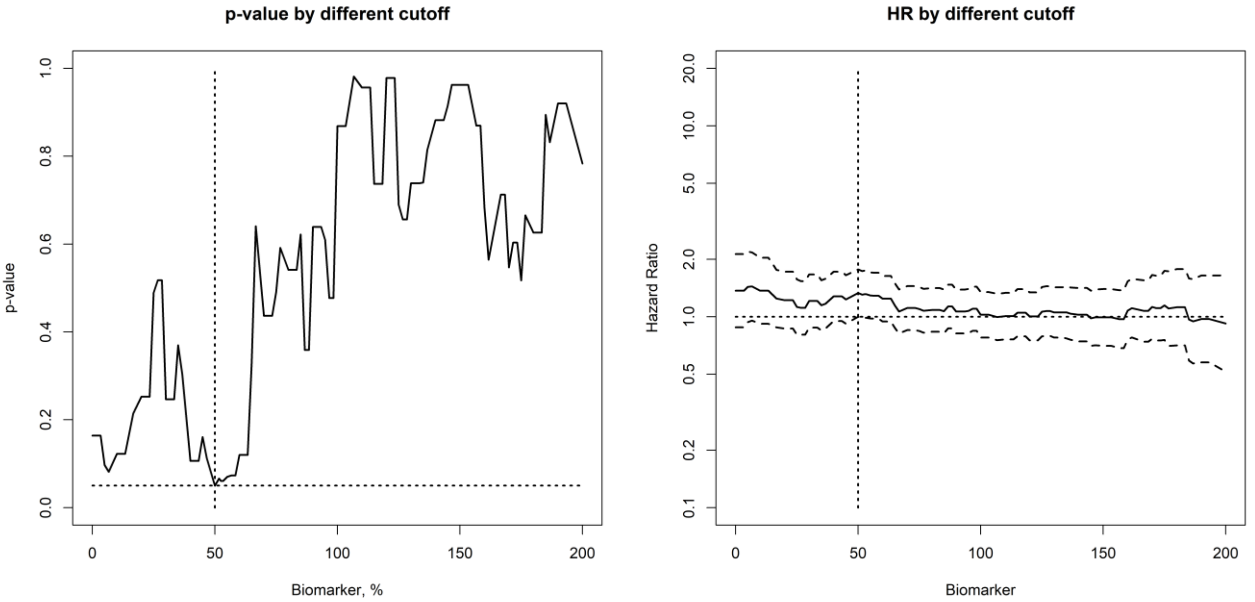

Figure S7. Best cut-off value of ADAM17 biomarker. (left) p value by different cut-off; (right) HR by different cut-off.
